# The evolution of an integrated ultrasound curriculum (iUSC) for medical students: 9-year experience

**DOI:** 10.1186/s13089-015-0035-3

**Published:** 2015-11-21

**Authors:** Richard A. Hoppmann, Victor V. Rao, Floyd Bell, Mary Beth Poston, Duncan B. Howe, Shaun Riffle, Stephen Harris, Ruth Riley, Carol McMahon, L. Britt Wilson, Erika Blanck, Nancy A. Richeson, Lynn K. Thomas, Celia Hartman, Francis H. Neuffer, Brian D. Keisler, Kerry M. Sims, Matthew D. Garber, C. Osborne Shuler, Michael Blaivas, Shawn A. Chillag, Michael Wagner, Keith Barron, Danielle Davis, James R. Wells, Donald J. Kenney, Jeffrey W. Hall, Paul H. Bornemann, David Schrift, Patrick S. Hunt, William B. Owens, R. Stephen Smith, Allison G. Jackson, Kelsey Hagon, Steven P. Wilson, Stanley D. Fowler, James F. Catroppo, Ali A. Rizvi, Caroline K. Powell, Thomas Cook, Eric Brown, Fernando A. Navarro, Joshua Thornhill, Judith Burgis, William R. Jennings, James B. McCallum, James M. Nottingham, James Kreiner, Robert Haddad, James R. Augustine, Norman W. Pedigo, Paul V. Catalana

**Affiliations:** Dorothea H. Krebs Endowed Chair of Ultrasound Education, University of South Carolina, School of Medicine, Columbia, SC 29208 USA

**Keywords:** Ultrasound, Education, Medical students, Integrated curriculum

## Abstract

Interest in ultrasound education in medical schools has increased dramatically in recent years as reflected in a marked increase in publications on the topic and growing attendance at international meetings on ultrasound education. In 2006, the University of South Carolina School of Medicine introduced an integrated ultrasound curriculum (iUSC) across all years of medical school. That curriculum has evolved significantly over the 9 years. A review of the curriculum is presented, including curricular content, methods of delivery of the content, student assessment, and program assessment. Lessons learned in implementing and expanding an integrated ultrasound curriculum are also presented as are thoughts on future directions of undergraduate ultrasound education. Ultrasound has proven to be a valuable active learning tool that can serve as a platform for integrating the medical student curriculum across many disciplines and clinical settings. It is also well-suited for a competency-based model of medical education. Students learn ultrasound well and have embraced it as an important component of their education and future practice of medicine. An international consensus conference on ultrasound education is recommended to help define the essential elements of ultrasound education globally to ensure ultrasound is taught and ultimately practiced to its full potential. Ultrasound has the potential to fundamentally change how we teach and practice medicine to the benefit of learners and patients across the globe.

**Electronic supplementary material:**

The online version of this article (doi:10.1186/s13089-015-0035-3) contains supplementary material, which is available to authorized users.

## Background

In 2006, the University of South Carolina School of Medicine introduced an integrated ultrasound curriculum (iUSC) across the 4 years of medical school for all students [1]. The first 4 years of this curricular experience were reported in 2011 [2]. Since that time significant expansion of the curriculum has been made and additional lessons have been learned about teaching ultrasound to medical students. The updated curriculum and important lessons learned will be presented in this 9-year review of the iUSC.

Interest in ultrasound education has increased dramatically in recent years. The number of citations reported in a PubMed search for “ultrasound education” has increased more than 2.5 times since 2006 from 351 to over 900 in 2014 [3]. More important than the absolute number of publications, however, is the quality of publications, the broad specialty and subspecialty interest in ultrasound education, and the thoughtful opinion pieces that have appeared in numerous journals [4–11]. Rising attendance at ultrasound education meetings such as those hosted by the Society of Ultrasound in Medical Education (SUSME) and the creation of ultrasound interest groups and meetings for students interested in ultrasound also attest to the broad-based and growing interest in undergraduate ultrasound education among educators and students [12–15].

There appear to be several factors contributing to this growing interest in ultrasound education. One is the mounting clinical evidence documenting the value of point-of-care ultrasound as an important diagnostic, clinical management, and procedural tool that results in improved patient safety and quality of care [16, 17]. Point-of-care ultrasound can be defined as ultrasound performed and interpreted by the clinician at the point of patient care. Point-of-care ultrasound examinations are not comprehensive ultrasound examinations but rather are focused examinations designed to answer specific clinical questions such as *Does this patient withright upper quadrant pain have a gallstone?* In addition, point-of-care ultrasound protocols have also been developed to provide practitioners with a systematic approach to more complicated differential diagnoses such as the BLUE protocol in the patient with acute respiratory failure or the multisystem approach of the RUSH protocol in the patient with hypotension and shock [18, 19]. Due to its clinical utility and safety profile since it does not use ionizing radiation, ultrasound is now considered the preferred or “First” imaging modality for a broad range of clinical scenarios [20–26].

Advances in ultrasound technology are also contributing to the growing interest in ultrasound. The image quality, system functionality, ease of use, and relatively low cost of portable ultrasound systems have made them more accessible for education and clinical practice. Portable ultrasound systems are available in a range of sizes including laptop, tablet, and pocket devices. There are also ultrasound transducers that plug into cell phones and tablets. The small size and light weight of portable ultrasound systems have made them accessible for use in virtually every patient care setting from the hospital and outpatient clinic to remote communities with limited medical services, to the site of a disaster, the battlefield, and even outer space [27–33].

Historically, students have had little ultrasound education and almost no hands-on scanning experience while in medical school. However, this is changing and more medical schools are introducing ultrasound into their curricula [34–46]. Considering the broad range of ultrasound applications that now cross nearly every specialty and subspecialty as well as the value of ultrasound as an active learning tool for many basic science and clinical subjects, an argument can be made that ultrasound should be seriously considered for inclusion as a core competency for all medical students.

## The curriculum

The University of South Carolina School of Medicine has a hybrid curricular structure with lectures, laboratories, small group learning, problem-based learning, clinical experiences, and six vertical curricula: geriatrics, nutrition, substance abuse, bioethics and professionalism, patient quality and safety, and ultrasound. The vertical curricula are integrated throughout the students’ 4 academic years (M1–M4). The integrated ultrasound curriculum is organized and managed by the multidisciplinary faculty of the ultrasound institute working closely with course and clinical clerkship directors.

A 4-year overview of the integrated ultrasound curriculum for medical students is presented in Table 1 and specifics of the curriculum are presented in Table 2. The delivery of ultrasound material and hands-on scanning laboratories are coordinated to complement and enhance the material being presented in various courses and clerkships. Multiple teaching modalities are used to deliver the curriculum, including 21 narrated web-based learning modules that cover a spectrum of important ultrasound topics from physics and instrumentation to basic cardiac and abdominal ultrasound [47]. Short videos of ultrasound scanning assignments prepare students for each hands-on laboratory and remain available to the students for review throughout their education. These videos are hosted on the University Ultrasound Institute YouTube channel for ready access [48]. In addition to learning modules and videos, both succinct laboratory handouts of curricular topics as well as more detailed handouts are made available to the students online for study and review.

**Table 1:** Overview of the ultrasound curricular across all 4 years: M1–M4

M1 year	M2 year	M3 year	M4 year
Anatomy	Introduction to clinical medicine—pathophysiology	Internal medicine clerkship	Emergency medicine ultrasound elective
Physiology	Pathology	Family medicine clerkship	Radiology elective with hands-on ultrasound
Neuroanatomy	Physical diagnosis^a^	Pediatrics clerkship	Ultrasound independent study month elective
Problem-based learning	Problem-based learning	Surgery clerkship	Capstone ultrasound course selective
		OB-GYN clerkship	Acting internship with ultrasound access
		Emergency medicine selective	
		Critical care selective	

^a^Two voluntary physical diagnosis small groups initiated 2014

**Table 2:** The integrated ultrasound curriculum (iUSC)

**First year (M1)**
Orientation week—before classes begin
1. Small group introductory ultrasound session
Basic instrumentation and knobology
Image orientation
Hands-on scanning of neck vessels
All education material available to students online throughout all four years: learning modules, videos, laboratory handouts and notes
Fall semester—in conjunction with gross anatomy
1. Introduction to cardiac ultrasound (laboratory session)
Parasternal long axis view (PLAX)—B-mode only; identification of heart chambers, valves, review screen orientation and image orientation marker location, knobology, depth, focus, frequency, gain adjustments
2. Neck ultrasound (laboratory session)
Carotid artery—B-mode and color flow mode—trace from common carotid to bifurcation, transverse and longitudinal views, basic principles of color flow Doppler
Internal jugular vein—B-mode and color flow mode; anatomic differences of internal jugular vein and carotid artery, shape, vessel wall, collapsibility, perform valsalva
Thyroid gland—B-mode; thyroid (both lobes and isthmus); echotexture, nodules, cysts, measurements, label structures, thyroid lobe volume estimation
3. Kidney and bladder ultrasound (laboratory session)
Urinary bladder—B-mode; identify bladder, measure bladder volume, note artifacts like posterior acoustic enhancement
Ureteric jets—color flow mode; test of total ureteric obstruction
4. Right and left upper quadrants (laboratory session)
Liver, gall bladder, right/left kidney, Morison’s pouch, diaphragm, and right costophrenic angle—B-mode
5. Introduction to musculoskeletal ultrasound—the knee (laboratory session)—B-mode
Anterior longitudinal suprapatellar view—patella, quadriceps tendon, femur, suprapatellar bursa
Suprapatellar tranverse flexed knee view—quadriceps tendon, femoral condyles, articular cartilage
Infrapatellar longitudinal view—patellar ligament, fat pad, tibia
Anisotropy artifact
6. Ultrasound OSCE—proper transducer selection, preset selection, probe orientation, scan and identify right kidney/liver/Morison’s pouch, left kidney/spleen, PLAX of the heart, carotid/internal jugular; student is also evaluated on their interaction with the standardized patient
Spring semester—in conjunction with physiology
1. Introduction to vascular ultrasound—vascular hemodynamics (laboratory)
Common carotid artery analysis
B-mode—transverse and longitudinal views
Color flow—direction of flow
Spectral Doppler/pulse wave—measure velocity, peak systolic velocity (PSV), end diastolic velocity (EDV), arterial and venous pulse wave forms
2. Heart ultrasound—hemodynamics (laboratory)
Apical 4 chamber view (B-mode and color flow mode)—wall motion, valve motion, cardiac cycle with color flow
3. Heart sounds and ECHO (laboratory)
Students work in pairs—one captures PLAX view showing both the aortic valve and mitral valve while other student listens with stethoscope and notes relationship of heart sounds and valve closure. Students then reverse roles
4. Cardiogenic shock—cardiac views: PLAX, apical 4-chamber, subcostal (laboratory session)
Cardiomypoathy—assess wall motion and shape of the left ventricle (LV) during cardiac cycle
Cardiac tamponade—assess for pericardial effusion, the right ventricle (RV) size and compression with cardiac cycle
Pulmonary embolism—assess for RV strain: size and compression with cardiac cycle; assess RV and right atrium (RA) for thrombosis
Spring semester—in conjunction with neuroanatomy
Brain and cranial nerves (presentation and demonstration)
Ultrasound measurement of optic nerve sheath diameter for assessment of increased intracranial pressure
Ultrasound assessment of direct and consensual pupillary light reflex
Ultrasound assessment of ocular movement for patients with marked orbital swelling
Spring semester—in conjunction with introduction to clinical medicine
Problem based learning ( small group discussion)—ultrasound relevant cases such 20 year old student who collapses during a basketball game—family history of sudden death and physical examination reveals a murmur—evaluation includes ECG, chest x-ray, and ECHO show hypertrophic cardiomyopathy
**Second year (M2)**
Fall semester—in conjunction with introduction to clinical medicine (ICM)
1. Cardiac ultrasound - standard cardiac views (laboratory session)
Parasternal long and short axis views, apical 4 and 5 chamber, subcostal; assess chambers, valves, wall thickness and motion
2. General abdomen (laboratory session)
Liver, gall bladder, kidneys, spleen, urinary bladder; identify structures and measure organ size
3. Abdominal aorta and inferior vena cava (IVC) assessment (laboratory session)
AAA screening; transverse and longitudinal, B mode, color flow and pulse wave, three measurements, characteristics that differentiate aorta from IVC
IVC—B mode and M mode, measurement and IVC collapsibility index
4. Lower extremity venous ultrasound (laboratory session)
Rule out deep venous thrombosis (DVT) in femoral, saphenofemoral junction, and popliteal vein— 2 point/level compression test, color flow Doppler, normal phasic venous flow, non-phasic venous flow, venous flow augmentation
Fall semester—in conjunction with pathology
Ultrasound images incorporated into lectures and small group clinicopathologic sessions to demonstrate pathologic and ultrasound correlates and enhance the transfer of pathology knowledge to the clinical diagnostic arena - many topics and images
Fall semester—physical diagnosis pilot (2014)
Small group physical diagnosis hands-on sessions—seventeen ultrasound components used to improve physical examination skills and enhance the accuracy of the physical examination—systems included: cardiovascular, pulmonary, abdomen, nervous system, ocular, and musculoskeletal
Spring semester—in conjunction with ICM
1. Female pelvic ultrasound—transabdominal (laboratory session)
Uterus, ovaries, pouch of Douglas, endometrium
2. Abdomen review and pancreas ultrasound—(laboratory session)
Upper abdominal vascular structures and transverse view of the pancreas—B mode—identify anatomical segments of the pancreas and normal echotexture
3. Ultrasound guided procedures (laboratory session with ultrasound phantoms)
Central venous access (Internal jugular vein)
Pleural effusion detection and pleurocentesis
Ascitic fluid/free fluid in peritoneal cavity - detection and paracentesis
4. Assessment of patient with undifferentiated shock (laboratory session)
RUSH protocol: rapid ultrasound for shock/hypotension—assess LV function, rule out pericardial effusion/tamponade, assess for RV strain from pulmonary embolus (PE), volume status from IVC size and dynamics, scan abdomen and pelvis for free fluid, assess lungs for pneumothorax and pulmonary edema, assess aorta for rupture, assess femoral vein for DVT
5. Ultrasound OSCE
Ultrasound OSCE station as part of an end-of-year comprehensive clinical skills OSCE. Each student conducts a focused history and physical examination on a standardized patients with one of three possible clinical scenarios then performs two corresponding ultrasound examinations: urinary bladder and abdominal aorta, renal/diaphragm and thyroid, cardiac and femoral vein
Spring semester—in conjunction with pathology
Ultrasound images incorporated into lectures and small group clinicopathologic sessions to demonstrate pathologic and ultrasound correlates and enhance the transfer of pathology knowledge to the clinical diagnostic arena—many topics and images
Spring semester—in conjunction with introduction to clinical medicine
Problem Based Learning ( small group discussion)—ultrasound relevant cases such as pregnancy with heart failure due to rheumatic heart disease—ECHO with mitral stenosis, chamber enlargement and “hockey-stick” mitral valve leaflet, lung ultrasound with B lines, fetal ultrasound
**Open ultrasound labs**
During the first two years (M1 and M2) open laboratory sessions are held weekly during a time when no other classes are scheduled. Students are encouraged to come in pairs or small groups and practice their ultrasound skills on each other. At least one ultrasound faculty member is available to help with scanning and answer questions
**Third year (M3)**
Clinical Rotations include clerkship specific ultrasound instruction—internal medicine, family medicine, pediatrics, surgery, obstetrics and gynecology. Instructional methods include image review sessions, bedside ultrasound rounds, independent and supervised patient scanning, simulation center ultrasound sessions, Ultrasound Institute scanning sessions, specialty and subspecialty ultrasound observation
Objective structured clinical examinations (OSCE) are administered at the end of the clerkship—below are some of the OSCEs that have been used over the nine years
1. Internal medicine
Thyroid ultrasound—patient with a “lump in the neck”, after the focused history and physical exam, each student must properly scan the thyroid and identify and measure a thyroid cyst
Septic patient who needs central line placement for intravenous access
2. Family and preventive medicine
Abdominal aortic aneurysm (AAA) screen—elderly patient with risk factors for AAA, student must discuss the procedure with the patient, perform the ultrasound examination, discuss results, and educate the patient about AAA
Musculoskeletal ultrasound in a patient with joint pain
3. OB/GYN
Two OSCE stations with previously captured images of findings that were covered with students during the rotation in observational and hands-on ultrasound learning sessions
OB ultrasound exam—patient is 27 weeks pregnant with a history of vaginal bleeding, student must perform an obstetrical ultrasound and determine fetal number, heart rate, placental location, and fetal position
4. Pediatrics
Assess soccer player who has “passed out twice during practice”—PLAX view with appropriate measurements for assessment of hypertrophic cardiomyopathy
Assess volume status/dehydration—9 year old with history of nausea/vomiting and poor oral intake, student must assess volume status using the aorta/inferior vena cava ratio
Interpretation of lung ultrasounds of a case of bacterial pneumonia with air bronchograms and pleural effusion
5. Surgery
Assess a trauma patient using the FAST exam (focused abdominal sonography for trauma)—each student must scan a patient for trauma and demonstrate Morison’s pouch, spleen/kidney interface, urinary bladder, sub-xiphoid view of the heart
One-week M3 selectives
Emergency medicine—supervised instruction and scanning of important emergency medicine ultrasound protocols, image review sessions, online emergency medicine ultrasound learning modules
Critical care medicine—supervised instruction and scanning in the intensive care unit for assessment of volume status, heart function, pneumothorax, and other important critical care scans
Ultrasound pocket devices on primary care clerkships (internal medicine, family medicine, pediatrics)
While on primary care clerkships students are issued pocket ultrasound devices for use and are encouraged to capture images from the heart, abdomen, and pelvis for submission and review at the end of the rotation
Ultrasound M3 Gate OSCE
All students at the end of the M3 year are required to complete a Gate OSCE that assessing a student’s readiness to progress to the M4 year. An ultrasound station is included
Assessment includes capturing a PLAX view of the heart and a longitudinal view of the inferior vena cava with a pocket ultrasound device to assess for heart function and volume status
Students must evaluate cardiac and IVC ultrasound loops on a laptop computer for overall heart function, pericardial effusion, and volume status
**Fourth year (M4)**
Four week emergency medicine ultrasound elective—online emergency medicine ultrasound learning modules, supervised instruction and scanning of emergency medicine patients and image review, a minimum of 10 eFAST examinations is required
Traditional Radiology elective with and ultrasound component that includes ultrasound learning modules, image review and “hands-on” ultrasound sessions focused primarily on guided procedure skill development
Ultrasound independent study month—work with ultrasound faculty and fellows to expand knowledge and skill in ultrasound. Includes scanning and ultrasound simulation, assisting with M1 and M2 ultrasound labs, participating in original research, and preparation of a 30 minute presentation on a ultrasound topic of their choosing
Two day capstone ultrasound course offered at the end of the 4th year—stresses ultrasound skills most important for students as they prepare for internship (ultrasound guided procedures, FAST exam, RUSH exam, lung ultrasound and soft tissue ultrasound to differentiate abscess and cellulitis)
M4 acting internships—students on acting internships have been offered pocket ultrasound devices when available

For hands-on ultrasound skills development, students have regularly scheduled required ultrasound laboratory sessions (5–6 per semester) in which they scan standardized patients under the supervision of a preceptor. Students also have opportunities to develop their scanning skills during “open” voluntary laboratory sessions held once or twice weekly during the semester. Students scan standardized patients and each other during these sessions and a preceptor is available to answer questions and assist with scanning. An ultrasound simulation manikin displaying real-time split-screen anatomy and simulated ultrasound images is available for student instruction during both the required laboratory sessions as well as the open laboratory sessions. The ratio of students to standardized patients during the ultrasound laboratory sessions is approximately four students per patient. Ultrasound-guided procedures are introduced to the students in the M2 year. Phantom models for ultrasound-guided procedures such as central and peripheral line access, paracentesis, and thoracentesis are available for students to practice their skills in the second, third, and fourth years of the curriculum.

### Neuroanatomy, pathology, and problem-based learning

Ultrasound images and demonstrations are used to complement courses without dedicated hands-on scanning sessions such as neuroanatomy, pathology, and introduction to clinical medicine problem-based learning (PBL) small groups.

During the neuroanatomy course in the M1 year, a case of a 60-year-old male with mental status changes, who is ultimately diagnosed with bacterial meningitis, is presented. An image of the optic nerve with increased nerve sheath diameter is key in identifying increased intracranial pressure that leads to the diagnosis. Discussion of causes and physical examination findings of increased intracranial pressure is followed by a live ultrasound demonstration of measurement of the optic nerve sheath diameter and assessment of pupillary light response and ocular movement.

In the two semester Pathology course in the M2 year, faculty use examples from the two “visually-rich” disciplines of pathology and ultrasonography in both large group lectures and small group case-based clinicopathologic sessions to help students better grasp the pathology being presented and transfer that knowledge to the clinical diagnostic arena [49]. The faculty have compiled a list of approximately eighty pathological entities with corresponding ultrasound images from appendicitis to cystic teratoma that are used to enhance student learning.

In small group PBL in the M1 and M2 years, illustrative ultrasound cases are used to help students acquire clinical knowledge and develop clinical reasoning skills. Such an example is the case of a 23-year-old male who collapses during a college basketball game. He has a family history of sudden death of an uncle at age 43. The case is one of hypertrophic cardiomyopathy with classic physical examination findings, chest X-ray, electrocardiogram (ECG), and echocardiogram (ECHO). The case presents many learning issues for the students from causes of syncope and sudden death in an apparently healthy young person, to the physical and laboratory findings of obstructive heart disease, and the genetics of heart disease.

## Clinical years

### Third-year (M3)

During the third-year of medical school five of the required seven clinical clerkships have additional ultrasound learning experiences for the students and have incorporated ultrasound into the end-of-rotation objective structured clinical examinations (OSCE). These clerkships include Internal Medicine, Family Medicine, Pediatrics, Surgery, and Obstetrics/Gynecology.

Emphasis is placed on ultrasound topics considered particularly important to the specific clerkship such as the Focused Assessment with Sonography in Trauma (FAST) examination on surgery, thyroid and heart assessment and central-line placement on internal medicine, and aortic aneurysm screening and musculoskeletal ultrasound on family medicine. Clerkship directors have created a variety of activities during the clinical rotations for students to continue developing their ultrasound knowledge and skills. These include bedside ultrasound rounds, independent and supervised scanning, image review sessions, scheduled time in the simulation center combining ultrasound with classic simulation such as birthing and central-line placement, specialty ultrasound observation, and scheduled time at the Ultrasound Institute reviewing clerkship pertinent ultrasound scanning protocols as well as training in more advanced scanning techniques.

In addition, when students rotate through the primary care clerkships of family medicine, internal medicine, and pediatrics they are issued a pocket ultrasound device for use during the clerkship. Students are encouraged to capture a variety of images of the heart, abdomen, and pelvis during these rotations and submit those images for review at the end of the rotation. Students also have a variety of 1-week selectives during the third-year and two of these selectives focus on ultrasound. One is a rotation in the intensive care unit and the other is in the emergency department. During these selectives the students have many opportunities to scan patients and review the images they capture with residents, fellows, and attendings. During these selectives, students enhance their scanning and interpretive skills and directly experience the impact ultrasound can have on patient care.

### Fourth-year (M4)

Although there are no ultrasound requirements in the fourth-year of medical school, there are multiple ultrasound opportunities for students to further their ultrasound knowledge and skill. These include for-credit electives such as a 4-week ultrasound independent study elective and an emergency medicine ultrasound elective. During the independent study month students review ultrasound material from years M1–M3, learn additional scanning protocols, assist with first- and second-year ultrasound laboratory sessions, and participate in research projects. Students have presented their research at regional and national meetings and some have had their research published. At the end of the independent study month, students are required to give a 20–30 min slide presentation on an ultrasound topic of their choosing. Students usually select topics relevant to their residency choice. For most of the students this presentation is their first ultrasound teaching presentation which they can use later during residency.

In the emergency medicine elective, students have access to online emergency medicine ultrasound learning modules. They receive supervised training of multiple scanning protocols and have the opportunity to perform many ultrasound examinations during the elective which are reviewed with upper-level residents, fellows, and attending physicians. They learn the extended-FAST (eFAST) examination and are expected to perform at least 10 such exams during the 4-week elective.

Ultrasound pocket devices are also made available for students during their required M4 acting internships and elective global medicine rotations. Fourth-year students who participate in the traditional M4 radiology elective get additional ultrasound exposure including hands-on practice sessions. Guided-procedure skills are emphasized during the radiology elective month. Toward the end of the fourth-year, students are offered a 2-day ultrasound experience during a Capstone period designated as review and preparation for residency. Important acute medical and surgical ultrasound protocols such as the rapid ultrasound for shock and hypotension (RUSH), e-FAST, and cardiopulmonary limited ultrasound examination (CLUE) protocols are covered [19, 50, 51]. An array of guided procedures is also reviewed and practiced.

Approximately 75 % of the students in the graduating class receive additional ultrasound training through one of these fourth-year options. It should be noted that specialty ultrasound electives and independent ultrasound study months can logistically be much easier to establish and get approved for the medical student curriculum than the introduction of ultrasound into core clinical rotations. Thus, this approach can be effective early in the process of introducing ultrasound into the curriculum even before ultrasound experiences in core clinical rotations are fully organized and approved.

## Assessment

Individual student learners are assessed regularly by several different testing methods and frequent feedback is obtained from the students about the various aspects of the curriculum.

### Student assessment

#### M1/M2

**Fig. 1:**
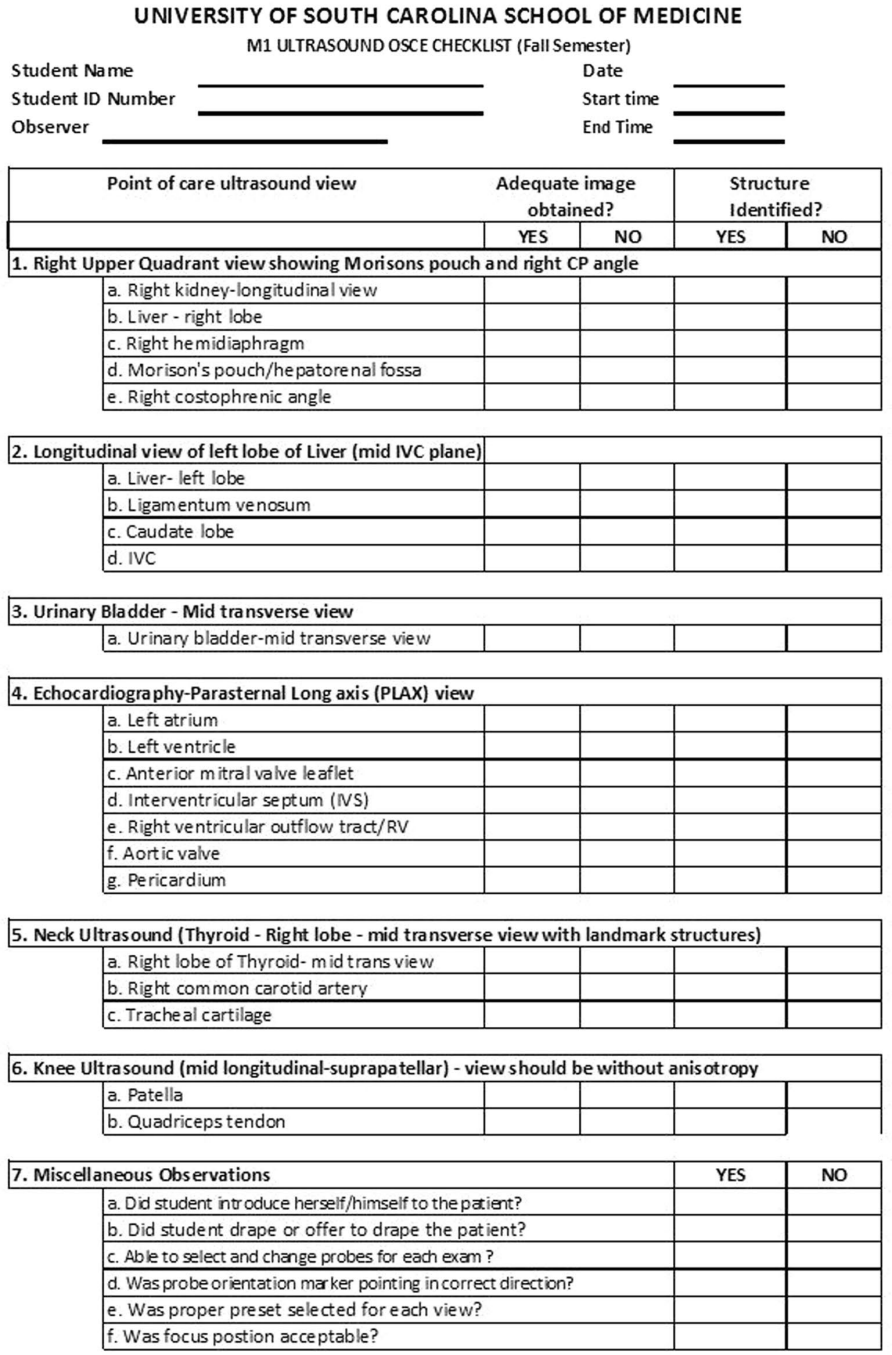
Ultrasound OSCE scoring checklist

During the first 2 years of medical school, students receive formative and summative evaluations by several methods. Assessment options include written multiple choice ultrasound questions as part of regular course examinations, image interpretation during practical examinations such as in gross anatomy, preceptor feedback during ultrasound laboratory sessions, image review from ultrasound laboratory sessions, small group preceptor evaluation, and OSCEs. OSCEs are used to assess the student’s ability to correctly use the ultrasound equipment, capture a pre-determined series of images, and identify structures in the images. Students are individually evaluated and are given a fixed period of time to complete the OSCE performed on a standardized patient. Each student is observed by an evaluator who completes an OSCE checklist like that in Fig. 1. Students are also evaluated on their interaction with the standardized patient. Students are expected to be courteous and professional in all standardized patient interactions. They are expected to introduce themselves to the patient, explain what will be performed, be attentive to the comfort and modesty of the patient, and thank the patient for his/her participation.

An OSCE has been given to first-year students at the end of the gross anatomy course since 2007. The overall structure of the OSCE has remained the same over the years but the content has been expanded such as the introduction of a musculoskeletal component in 2011. Students have performed exceptionally well on the OSCE over the course of the program as can be seen from Table 3. Yearly means of each class show consistently high scores with an overall 8-year mean of 96.1 %.

**Table 3:** Student OSCEs results—percent correct

	2007–2008	2008–2009	2009–2010	2010–2011	2011–2012	2012–2013	2013–2014	2014–2015	Mean
M1									
Mean OSCE score (%)	98.2	97.4	95.6	97.2	96.2	95.4	93.9	94.9	96.1
Range (%)	78–100	64–100	64–100	61–100	75–100	74–100	34–100	34–100	61–100
M2									
Mean OSCE score (%)	97.2	98.0	91.0	96.2	98.5^a^	97.7^a^	93.5^a^	95.7^a^	96.0
Range (%)	71–100	83–100	50–100	80–100	91–100	82–100	63–100	53–100	72–100

^a^M2 ultrasound OSCE incorporated into end-of-year comprehensive clinical skills OSCE

Up until 2011, the M2 students were assessed with a similar OSCE format as the M1s. They also have performed well on this OSCE with a 4-year average of 95.6 %. In 2011, a new format for M2 ultrasound OSCE was introduced. Instead of a free-standing ultrasound-only OSCE, ultrasound competency assessment was incorporated into an end-of-year Introduction to Clinical Medicine (ICM) comprehensive clinical skills OSCE.

Students began the evaluation process with a typical patient encounter that required a focused history and physical examination as one might complete for a patient with abdominal pain or shortness of breath. The student would then proceed to a second station for Direct Observation of Procedural Skills (DOPS) and would be required to perform two ultrasound scanning protocols relative to the patient’s chief complaint. For example, in a patient with shortness of breath the student would be required to perform a parasternal long axis view of the heart to assess cardiac function and a deep venous thrombosis compression test of the femoral and saphenous veins for possible thrombosis. These are scanning protocols the student learned during the M1 and M2 years. Students had to be prepared to perform two of six potential ultrasound scans because they did not know ahead of time which clinical scenario they would be given. In addition to performing the ultrasound scan and interpreting the image, students were also required to answer several clinically relevant questions such as the relative size of the right and left ventricles in the setting of a large pulmonary embolus. Students have performed well on this new OSCE format with average scores of 96.4 %.

## Ultrasound image review portal

**Fig. 2:**
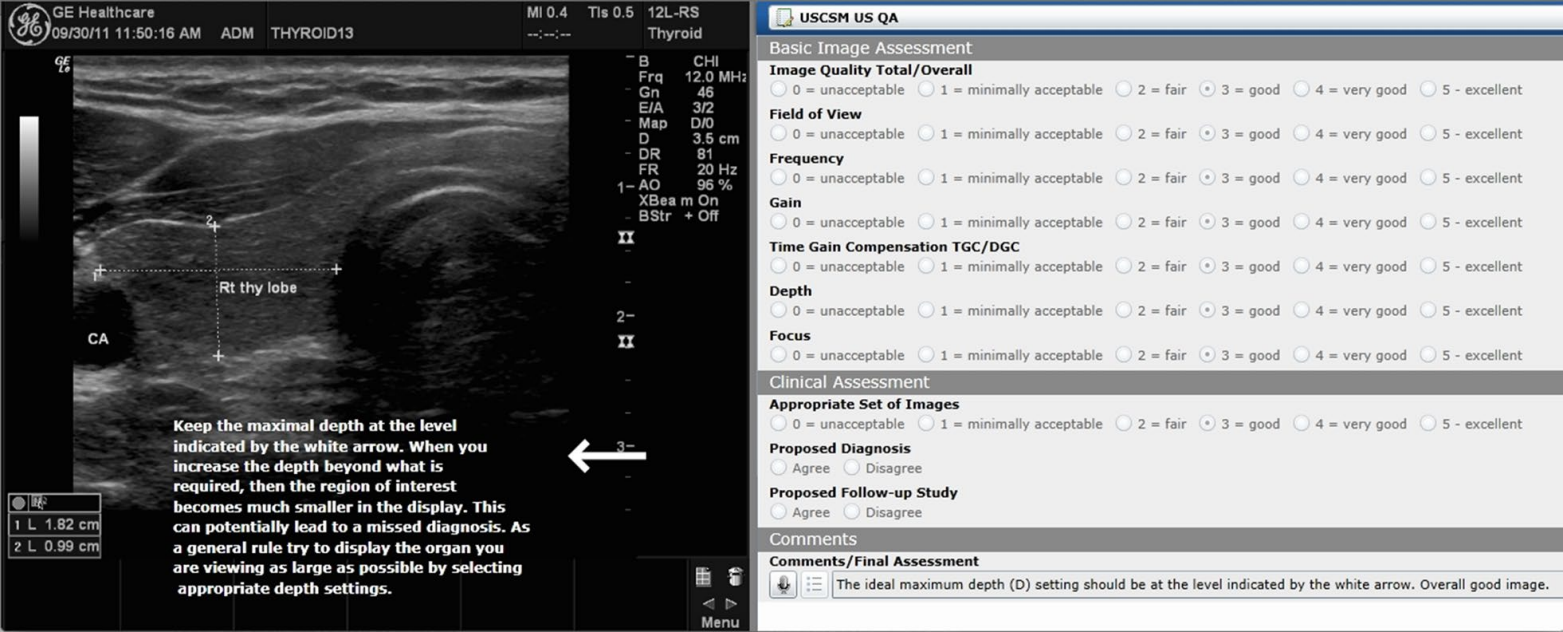
Student ultrasound image evaluation via cloud-based review portal

For scheduled ultrasound laboratory sessions during the M1 and M2 years, students must capture and label relevant ultrasound images and submit them for review via a cloud-based ultrasound image review portal. Each image is reviewed by an Ultrasound Institute faculty member, fellow, or sonographer and formative feedback is given to the student. In Fig. 2, the two components of the image evaluation are displayed. Narrative comments and indicators from the evaluator are placed directly on the ultrasound image. In the case displayed, the evaluator recommended that the student adjust the “depth” to capture more of the target structure which in this case is the thyroid. The evaluator’s recommendation is displayed on the image at the level of the arrow to show the student the best place to set the depth to allow better visualization of the thyroid and decrease dead space deep to the thyroid. Also seen in Fig. 2 is the standardized evaluation form used to assess the overall quality of the image and specific image characteristics such as gain and focus using a six point Likert scale.

### M3 assessment

As previously noted, ultrasound stations have been incorporated into end of clerkship OSCEs. In addition, in 2012 an ultrasound station was introduced into the M3 Gate OSCE that assesses a student’s readiness to progress to the M4 year. The initial ultrasound OSCE station was created to assess the student’s competency to perform a parasternal long axis cardiac view for overall heart function and to estimate volume status by means of a longitudinal view of the inferior vena cava. In 2013, an image interpretation component was added. After students captured ultrasound loops of the heart and inferior vena cava in a standardized patient using a pocket ultrasound device (Vscan), they were then required to assess a series of 10 ultrasound loops for heart function, pericardial effusion, and volume status on a laptop computer in the examination room. Students have performed well on this ultrasound OSCE component of their Gate examination. They have been able to capture quality images of the heart and inferior vena cava and the average image interpretation score in 2014 was 86 %.

Also during the third-year, students are given feedback on the images they submit as part of the primary care clerkship pocket ultrasound program. These images generally include views of the heart, inferior vena cava, abdominal aorta, gallbladder, and urinary bladder with ureteric jets. The overall quality of the images submitted have been between *good* and *very good* on a scale of *unacceptable*, *minimally acceptable*, *fair*, *good*, *very good*, and *excellent*.

## Course program evaluation

M1 and M2 students complete anonymous online course evaluations at the end of each semester. The average response rate for these evaluations is above 90 %. Table 4 is a summary of course evaluations over the 9-year period of the iUSC. Likert scores and percentage of students responding with *agree* or *strongly agree* to questions have been consistent from year-to-year. For the M1 and the M2 classes, 93 % of the students feel the ultrasound curriculum has enhanced their overall medical education. Just over 90 % of students feel ultrasound has allowed for increased clinical correlation with basic science instruction and has enhanced their understanding of the physical examination. Roughly 75 % of the students would like to see more ultrasound in the curriculum. The online surveys conclude with an opportunity for student comments. The overwhelming majority of these comments have been positive such as “Ultrasound was a great way to reinforce the information we learned in anatomy” and “Great addition to the curriculum.”

**Table 4:** Summary of student curriculum evaluations 2006–2014

Questions	Mean Likert score^a^	Mean % responding agree or strongly agree
M1 class		
1. The use of ultrasound in gross anatomy has enhanced my ability to learn basic anatomy	4.16	84.7 %
2. The use of ultrasound in physiology has enhanced my ability to learn basic physiology	3.83	71.4 %
3. I found the scheduled hands-on laboratory sessions with standardized patients helpful in learning ultrasonography	4.43	92.1 %
4. I found the open laboratory sessions used to practice scanning each other helpful in learning ultrasonography	4.31	80.2 %
5. I found the overall educational experience in ultrasound enhanced my medical education	4.44	93.2 %
6. I would like to see more ultrasound in the curriculum	4.17	79.4 %
M2 class		
1. The use of ultrasound in the Introduction to Clinical Medicine (M2) has allowed for increased clinical correlation with basic science instruction	4.27	90.3 %
2. Ultrasound has enhanced my understanding and skills of the physical exam	4.25	90.2 %
3. I found the scheduled hands-on laboratory sessions with standardized patients helpful in learning ultrasonography	4.48	91.2 %
4. I found the open laboratory sessions used to practice scanning each other helpful in learning ultrasonography	4.11	75.0 %
6. I found the overall educational experience in ultrasound enhanced my medical education	4.44	93.1 %
7. I would like to see more ultrasound in the curriculum	4.03	70.5 %

^a^Scale: 1 = Strongly disagree; 2 = disagree; 3 = neither agree nor disagree; 4 = agree; 5 = strongly agree

From the comment section also come constructive feedback and suggestions for improving the curriculum. Many of the suggestions are incorporated into the curriculum the following year, for example, the students’ request for more open laboratory time and requests for specific organ system scanning such as the musculoskeletal system. The comments also provide important feedback that can be shared with the administration to validate the value of the ultrasound curriculum. Examples such as “Ultrasound is the reason I chose to come to this medical school” and “I feel this ultrasound experience gives me an advantage in caring for my future patients” are shared with the Dean’s office. Based on this feedback, the medical school applicant interview day now includes a tour of the Ultrasound Institute and ultrasound is highlighted in the school’s promotional videos.

M3 students complete an online survey late in the spring of the third-year. The majority of students report that ultrasound has enhanced their education during clinical rotations and more recently report positive experiences related to the pocket devices added in 2011, especially with respect to clinical diagnosis and the physical examination. Students have reported establishing diagnoses such as pericardial effusion or gallstones with these pocket devices that had not been appreciated by the patient care team prior to the student scanning the patient.

Fourth-year experiences in ultrasound including independent study, the emergency medicine elective, the radiology elective, and the Capstone selective have also received very positive evaluations from the students.

## A physical diagnosis pilot

In 2014, a physical diagnosis ultrasound pilot study was conducted with two of twelve small groups in the Introduction to Clinical Medicine course. Seventeen ultrasound components were used to expand the physical examination, improve its accuracy, and serve as feedback to improve the traditional physical examination skills of the students. This last objective is particularly important because some of the resistance to incorporating ultrasound into the medical student curriculum has been the fear that physical examination skills will not be adequately learned by students as they may rely on ultrasound instead. It has also been pointed out that ultrasound will not be available in all practice settings.

In the heart sounds video (Additional file 1: Video S1) are instructions for an exercise designed to improve auscultation skills. Students work in pairs with one student performing an ECHO while the other student listens with a stethoscope at various locations on the chest. While listening, the student watches the real-time ECHO and matches the heart sounds with the cardiac cycle and valve closure. We have created several additional videos such as aortic insufficiency (Additional file 2: Video S2) in which one can hear the first or S1 heart sound with closure of the mitral valve and the second or S2 heart sound with closure of the aortic valve followed by the murmur of aortic insufficiency. Aortic Insufficiency with Doppler (Additional file 3: Video S3) visualizes the murmur of aortic insufficiency with color Doppler. These videos were created by simultaneously recording the ECHO and the corresponding heart sounds. Students have reported this auscultation exercise and the ECHOs with heart sounds helpful in learning the heart examination.

Our limited results from the pilot study found students to be enthusiastic about learning both traditional physical examination skills and ultrasound. Additionally, 100 % of the students “agreed” or “strongly agreed” that “Ultrasound enhanced my ability to learn the traditional physical examination.”

## Lessons learned

Some of the lessons learned in the first 4 years of the integrated ultrasound curriculum still hold true and are worth summarizing briefly [2].

- Start small, and work closely with course and clerkships directors. It is important not to overwhelm faculty or students with too much new material too quickly. The ultrasound components introduced should be well thought out and coordinated with the rest of the curriculum.
- Introduce ultrasound early in the curriculum and provide opportunities for students to practice outside of scheduled laboratories. Not all students learn at the same pace and some will want to learn more than what is required.
- There is no need to create all learning material in-house. Very good ultrasound educational material has been created in recent years and much of it is open access.
- Identify and support faculty champions and gather student feedback regularly. Ultrasound can be an academic niche for faculty and the students as the curricular users provide essential input.
- An ultrasound program will attract good students. It incorporates state-of-the-art technology and provides early clinical exposure. Provide feedback to the school administration and advertise the program.

Additional lessons have been learned during the last 5 years of the curriculum and are best appreciated in the context of the competency-based medical education model which will be the prevailing education model for the foreseeable future [52, 53].

- Provide as much flexibility in learning ultrasound as possible both with respect to time and modalities of learning. This will allow more self-paced, self-directed, and individualized learning. Most of the didactic lectures on ultrasound have been eliminated from our curriculum and have been replaced by online learning modules, videos, and open laboratory sessions for scanning live models and ultrasound simulation.
- Outcomes should be observable and measurable and assessment should be formative, summative, and include measurement of application of knowledge and skill. An ultrasound image portal has been implemented for objective evaluation of acquired images and as a means to assess student progress and provide feedback across courses and clerkships. This has added considerable flexibility and convenience for students and faculty. Additional OSCEs have been introduced into the curriculum including a M2 end of pre-clinical training OSCE and a M3 Gate OSCE both of which include assessment of ultrasound knowledge and skill in the context of common clinical scenarios.
- In physical diagnosis, the immediate feedback ultrasound provides has great potential to help students develop their skills of auscultation, palpation, and percussion. Ultrasound can also be used as an objective measure of bedside physical examination skills such as estimating liver size or correctly identifying a murmur.

An illuminating observation has been made over these 9 years of the iUSC and that is the remarkable power of ultrasound to bring together basic scientists, clinicians, and educators in ways that enhance integration of the overall medical student curriculum both horizontally and vertically. A common medical problem such as heart failure can be traced with ultrasound from the normal anatomy and physiology of the heart, to the pathophysiology and pathology of heart failure, to a better understanding of pharmacologic intervention, to enhancement of the physical examination of the heart, and finally to real-life diagnosis and management of heart failure in the clinical arena. Ultrasound also brings the teacher and the learner back to the bedside where the art and the science of medicine merge and are best learned. This broad multidisciplinary integration also means that there are many points in the curriculum where ultrasound can be taught and thus, less ultrasound curricular time is needed for any one discipline or specialty.

## Conclusions and future directions

Based on 9 years of experience with an integrated ultrasound curriculum, the value of ultrasound as an active learning tool, as a platform for curricular integration, and as an important clinical skill is clear. In addition, students can learn basic ultrasound well and have embraced it as an important component of their future practices. Ultrasound by its very nature fits nicely into a competency-based model of medical education. Ultrasound curricula can be designed with flexibility and objectivity that allow self-directed and self-paced learning. Ultrasound has important clinical applications that can be assessed across many specialties and clinical settings and will ultimately improve patient care.

Although our integrated ultrasound curriculum has matured significantly over the past 9 years, we still consider it a work in progress. Plans are underway to add new content and continue movement toward a fully competency-based medical education model with a student E-portfolio to track student progress in the attainment of curricular milestones, competencies, and entrustable professional activities as they are further defined. Although it is difficult to conduct well-controlled education studies with large numbers of students over extended time periods due to the many variables involved, an E-portfolio tracking system should facilitate such studies. In addition, small but well-controlled clinically relevant studies of student application of ultrasound will continue to be part of the ultrasound program [54, 55].

Plans are also underway to develop small group clinical cases with ultrasound components in medical pharmacology suitable for the emerging “flipped classroom” approach to medical education. More ultrasound education will be introduced for faculty who will not be performing ultrasound scans but need to acquire a basic understanding of ultrasound terminology, applications, and image interpretation. This will allow this non-scanning faculty (NSF) to more fully participate in medical student education. This model has been successfully introduced into residency education [personal communication David M. Tierney]. Automated image assessment of individual learners using ultrasound simulation is also planned which will allow tracking of the progress of each student as well as the effectiveness of the curriculum from analysis of all students’ simulation data.

It appears that interest in ultrasound in medical education will continue to grow and more schools nationally and globally will integrate ultrasound into their curricula. At present there is no consensus curriculum for ultrasound education. There are two organizations, however, that have joined forces to conduct an international consensus conference on ultrasound education. The Society of Ultrasound in Medical Education (SUSME) is an academic society committed to fostering collaboration and communication across multiple disciplines to promote ultrasound education globally [56]. SUSME has hosted three conferences on Anatomy and Physiology Ultrasound Education and with the World Interactive Network Focused on Critical Ultrasound (WINFOCUS) has hosted three World Congresses on Ultrasound in Medical Education [57]. WINFOCUS has been a world leader in promoting point-of-care ultrasound practice and has hosted multiple International Consensus Conferences to better define ultrasound use and establish standards in critical areas of medical practice [58–60]. Together these two organizations are well-positioned for the important task of undertaking a global consensus process among ultrasound education and practice leaders from multiple disciplines, organizations, and regulatory bodies to help define essential elements of ultrasound education.

The future appears very bright for ultrasound in medical education and clinical practice. As healthcare professionals and academicians we have a responsibility to ensure ultrasound education and practice are done well and are held to the high standards of our profession. If we are successful in that charge, ultrasound has the potential to fundamentally change how we teach and practice medicine to the benefit of learners and patients across the globe for decades to come.

## Additional files

10.1186/s13089-015-0035-3 Heart sounds.
10.1186/s13089-015-0035-3 Aortic insufficiency.
10.1186/s13089-015-0035-3 Aortic insufficiency with Doppler.
